# Anti-cancer effects of Rhizoma Curcumae against doxorubicin-resistant breast cancer cells

**DOI:** 10.1186/s13020-018-0203-z

**Published:** 2018-08-29

**Authors:** Zhangfeng Zhong, Haibing Yu, Shengpeng Wang, Yitao Wang, Liao Cui

**Affiliations:** 1 Guangdong Key Laboratory for Research and Development of Natural Drugs, Guangdong Medical University, Zhanjiang, Guangdong China; 2 State Key Laboratory of Quality Research in Chinese Medicine, Institute of Chinese Medical Sciences, University of Macau, Macao, China; 3 School of Public Health, Guangdong Medical University, Dongguan, Guangdong China

**Keywords:** Rhizoma Curcumae, Multidrug resistance, Breast cancer, ABC transporters, Furanodiene

## Abstract

**Background:**

Chemotherapy is a primary approach in cancer treatment after routine surgery. However, chemo-resistance tends to occur with chemotherapy in clinic, resulting in poor prognosis and recurrence. Nowadays, Chinese medicine may shed light on design of new therapeutic modes to overcome chemo-resistance. Although Rhizoma Curcumae possesses anti-cancer activities in various types of cancers, the effects and underlying mechanisms of its bioactive components against chemo-resistance are not clear. Therefore, the present study aims to explore the potential effects of Rhizoma Curcumae on doxorubicin-resistant breast cancer cells.

**Methods:**

The expression and function of ABC transporters in doxorubicin-resistant MCF-7 breast cancer cells were measured by western blotting and flow cytometry. Cell viability was detected using MTT assay. The combination index was analyzed using the CalcuSyn program (Biosoft, Ferguson, MO), based on the Chou–Talalay method.

**Results:**

In our present study, P-gp was overexpressed at protein level in doxorubicin-resistant MCF-7 cell line, but short of MRP1 and BCRP1. Essential oil of Rhizoma Curcumae and the main bioactive components were assessed on doxorubicin-resistant MCF-7 cell line. We found that the essential oil and furanodiene both display powerful inhibitory effects on cell viability, but neither of these is the specific inhibitor of ABC transporters. Moreover, furanodiene fails to enhance the efficacy of doxorubicin to improve multidrug resistance.

**Conclusion:**

Overall, our findings fill the gaps of the researches on chemo-resistance improvement of Rhizoma Curcumae and are also beneficial for Rhizoma Curcumae being developed as a promising natural product for cancer adjuvant therapy in the future.

**Electronic supplementary material:**

The online version of this article (10.1186/s13020-018-0203-z) contains supplementary material, which is available to authorized users.

## Background

Chemotherapy is regarded as one of adjuvant therapy after a routine surgery and being the primary approach for various cancer types [1–3]. However, many obstacles, including low efficacy and side effects, especially for chemo-resistance, still exist in cancer patients undergoing chemotherapy. There are a lot of strategies overcoming chemo-resistance, such as targeting ATP-binding cassette (ABC) transporters [4, 5], inducing cell apoptosis [6], inhibiting DNA repair [7], regulating metabolic reprogramming [8, 9], or applying combination therapy [10]. The role of ABC transporters is found to be closely related with chemo-resistance, thereby leading to poor prognosis and tumor recurrence in clinic [11]. Because of the expressions of ABC transporters, efflux pump decreases intracellular accumulation of drugs, then therapeutic concentrations of effective agents are reduced [12]. A thorough mechanisms of ABC transporters in chemo-resistance are still ongoing, and some typical proteins have been hot topics for a long time, including P-glycoprotein 1 (P-gp, MDR1, or ABCB1) [13], multidrug resistance-associated protein 1 (MRP1) [14], and ATP-binding cassette sub-family G member 2 (ABCG2 or BCRP) [15, 16].

**Fig. 1:**
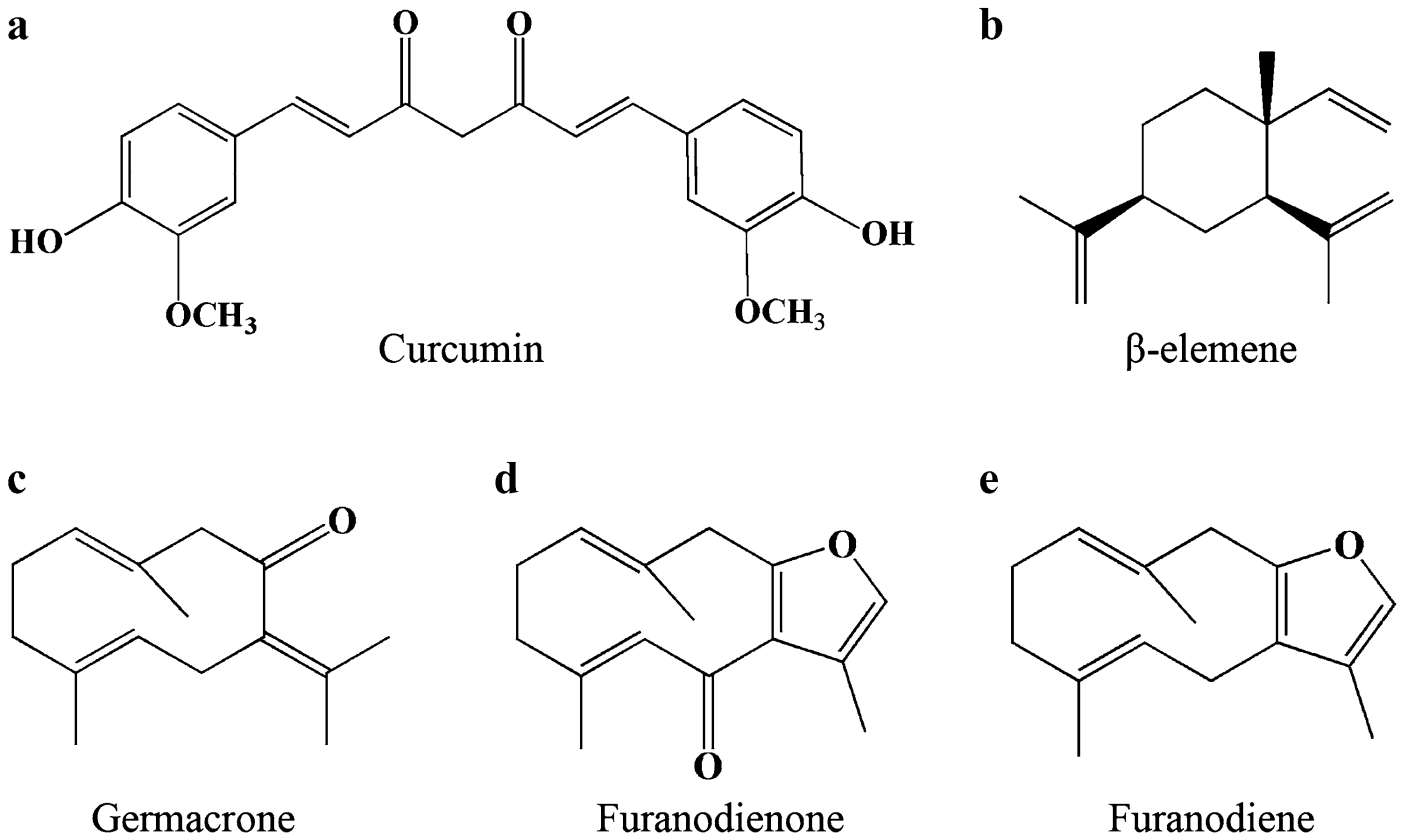
Chemical structures of the main compounds derived from Rhizoma Curcumae. **a** Curcumin. **b** β-elemene. **c** Germacrone. **d** Furanodienone. **e** Furanodiene

Overcoming chemo-resistance is a big challenge to chemotherapy. Natural products are rich sources of bioactive constitutes reversing cancer multidrug resistance, enhancing efficacy of anti-cancer agents, and decreasing side effects [9, 17, 18]. Currently, growing evidence show that Rhizoma Curcumae exhibits therapeutic value in overcoming chemo-resistance. The fractionated extracts of Rhizoma Curcumae improve the sensitivity of doxorubicin-resistant MCF-7 breast cancer cells to doxorubicin by blocking P-gp activity and down-regulating P-gp expression [19]. Furthermore, the fraction of CH_2_Cl_2_ extract is much more effective than that of EtOAc extract to restore the sensitivity of chemo-resistant MCF-7 cells to anti-neoplastic agents [20]. Meanwhile, several pure compounds (as shown in Fig. 1) isolated from Rhizoma Curcumae also have been reported to possess anti-cancer activities in multidrug resistant cancer cells. In detailly, curcumin inhibits viability of chemo-resistant breast cancer cells in an ER-independent manner and reverses multidrug resistance through ABC transporters [21]. β-elemene enhances cytotoxic effect of doxorubicin on doxorubicin-resistant MCF-7 breast cancer cells, which is related to increased doxorubicin accumulation and decreased Bcl-2 expression [22]. Germacrone reverses multidrug resistance through inducing cell apoptosis by down-regulation of Bcl-2 and up-regulation of p53 and Bax. In addition, germacrone significantly reduces P-gp expression in multidrug resistant breast cancer cells [23].

Combination therapy is a treatment combining two or more therapeutic agents, aiming to improve disease-specific symptoms and overall survival. During and after cancer treatment, combination therapy potentially reduces chemo-resistance and provide therapeutic anti-cancer benefits simultaneously [24, 25]. Meanwhile, a variety of components derived from Chinese medicinal herbs are undergoing extensive researches of combination treatments in overcoming multidrug resistance to enhance efficacy [26, 27]. Therefore, it is urgent to develop an integrative approach to cancer care, when combination therapy meets Chinese medicinal herbs [28, 29]. To the best of our knowledge, there is no report of furanodiene or essential oil from Rhizoma Curcumae exhibiting anti-cancer effects in chemo-resistant cancer cells by means of ABC transporters regulation. Therefore, our present study identified anti-cancer effects of those bioactive constituents from Rhizoma Curcumae initially and explored the related mechanisms in doxorubicin-resistant MCF-7 breast cancer cells.

## Methods

The Minimum Standards of Reporting Checklist contains details of the experimental design, and statistics, and resources used in this study (Additional file 1).

### Chemical and reagents

The Roswell Park Memorial Institute-1640 (RPMI-1640) culture medium were purchased from Gibco (Maryland, USA). Fetal bovine serum (FBS), phosphate-buffered saline (PBS), penicillin-streptomycin (PS), and 0.25% (w/v) trypsin/1 mM EDTA were obtained from Invitrogen (Carlsbad, USA). 3-[4, 5-Dimethyl-2-thiazolyl]-2, 5-diphenyltetrazolium bromide (MTT) and Calcein AM were obtained from Molecular Probes (Eugene, USA). Doxorubicin (DOX) and Rhodamine 123 were supplied by Sigma-Aldrich (St. Louis, USA). Furanodiene and furanodienone were purchased from National Institutes for Food and Drug Control. Essential oil was obtained as our previous report [30]. Radioimmunoprecipitation assay (RIPA) lysis buffer and primary antibodies against P-gp, MRP1, and BCRP1 were obtained from Santa Cruz (Santa Cruz, USA). Primary antibodies against β-actin, as well as the secondary antibodies were purchased from Cell Signaling (Danvers, USA).

### Cell culture and drug treatment

MCF-7 cell line was obtained from the ATCC (Manassas, USA) and was cultured as previously reported [31]. To induce doxorubicin-resistant cancer cell line, MCF-7 cells were cultured with RPMI1640 medium containing fetal bovine serum (10%), penicillin (100 units per mL), and streptomycin (100 μg/mL), at 37 °C in a humidified atmosphere of 5% CO_2_ in air. Doxorubicin-resistance was established by stepwise exposure to increased concentrations of doxorubicin as described previously [32]. The stock solutions of essential oil (100 mg/mL), furanodiene (100 mM), furanodienone (100 mM), and doxorubicin (2 and 100 mM) dissolved in DMSO were diluted to different concentrations as required.

### Cell viability assay

Cell viability was performed using MTT assay as described previously [33]. Briefly, exponentially growing cells were seeded in 96-well plates at a density of 2 × 10^4^/well and allowed to attach overnight. Following the required incubation period, cell viability was determined by adding MTT working solution (100 µL/well, 1 mg/mL). The absorbance values at 570 nm were recorded using SpectraMax M5 microplate reader (Molecular Devices, Silicon Valley, USA).

### Western blotting assay

Western blotting assay was performed according to previous studies [34]. Briefly, exponentially growing cells were seeded in culture dish (100 mm) at a density of 2 × 10^6^/dish and allowed to attach overnight. After the required treatments, cells were harvested, and the total proteins were extracted with RIPA lysis buffer. Equal amounts of total proteins were separated by appropriate SDS-PAGE followed by transferring onto a PVDF membrane. After blocking with 5% non-fat milk, the membrane was incubated with specific primary antibodies (1:1000 v/v) and the corresponding second antibodies (1:2000 v/v), respectively. The specific protein bands were visualized with an Amersham™ ECL™ advanced western blotting detection kit (GE Healthcare Life Sciences, UK).

### P-gp expression assay

P-gp expression was evaluated using the antibody of P-glycoprotein conjugated FITC (BD Biosciences, San Jose, USA) as described previously [35]. Cells were seeded into 6-well plates at a density of 2 × 10^5^/well, followed by the required drug treatments. The cells were harvested and incubated with 100 µL of P-gp-FITC antibody dye-loading buffer at 37 °C for 30 min protected from light. The mean fluorescence intensity of FITC was detected using a flow cytometry (BD FACS Canto™, BD Biosciences, San Jose, USA). And the results were analyzed by FlowJo software (TreeStar, Ashland, OR, USA).

### P-gp function assay

Rhodamine 123 and Calcein AM were applied to determine the activity of P-gp as described previously [35]. Cells were seeded into 6-well plates at a density of 2 × 10^5^/well, followed by the required drug treatments. 100 µL of Rhodamine 123 or Calcein AM dye-loading solutions were added to each well and incubated at 37 °C for 30 min protected from light. The cells were harvested, and intracellular fluorescence was detected using a flow cytometry and analyzed by FlowJo software.

### Doxorubicin uptake assay

Cells were seeded in 6-well plates at a density of 2 × 10^5^/well and were treated with different concentrations of test agents in the presence of doxorubicin. Following a 2-h incubation, the cells were washed and re-suspended in dye-free culture medium. The doxorubicin uptake was assessed using flow cytometry and the results were also analyzed by FlowJo software.

### Statistical analysis

All data represent the mean of three independently performed experiments, plus or minus standard deviation or standard error of the mean. The significance of intergroup differences was evaluated by one-way ANOVA using the GraphPad Prism software (GraphPad Software, USA). Newman–Keuls multiple comparison tests were performed for *post hoc* pairwise comparisons. *p*-values less than 0.05 were considered as significant.

## Results

### Establishment and characterization of doxorubicin-resistant MCF-7 breast cancer cell line

**Fig. 2:**
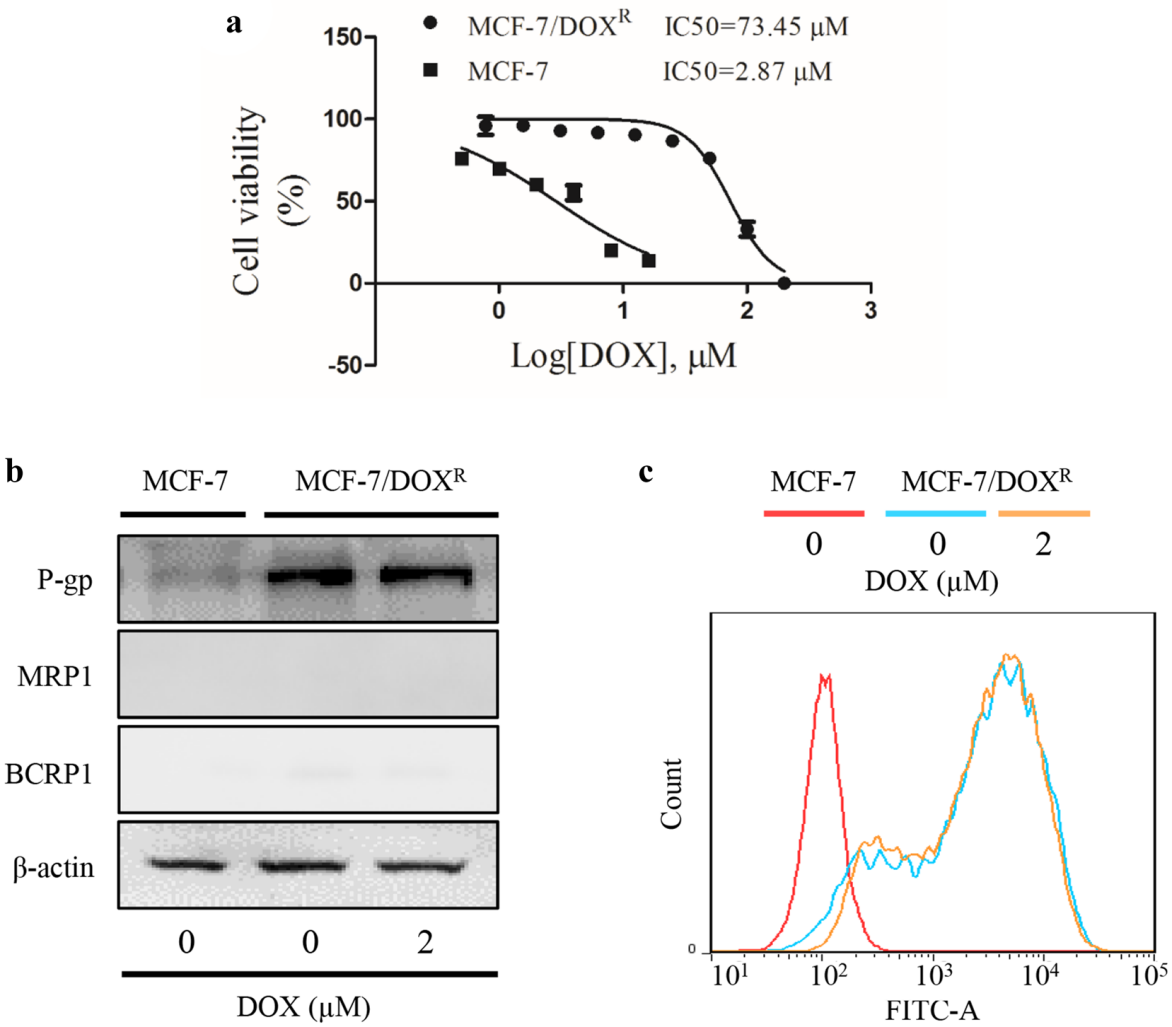
Establishment and characterization of the doxorubicin (DOX)-resistant MCF-7 breast cancer cell line. Doxorubicin-resistant MCF-7 breast cancer cell line (MCF-7/DOX^R^) was established by a stepwise exposure of MCF-7 cells to increasing concentrations of doxorubicin (DOX). Cells were treated with DOX for 48 h. **a** Cell viability was tested using MTT assay, represented by percentage of control. **b** Protein expression was evaluated using western blotting assay. **c** The expression alterations of P-gp were confirmed by FITC-P-gp antibody staining using flow cytometry

Doxorubicin-resistant MCF-7 breast cancer cell line was established by a stepwise exposure of MCF-7 cells to increasing concentrations of doxorubicin. Cell viability was tested by MTT assay after a 48-h treatment of doxorubicin. Our results show that doxorubicin-resistant MCF-7 cells are resistant to doxorubicin with an IC50 value of 73.45 µM. And MCF-7 cells are sensitive to doxorubicin with an IC50 value of 2.87 µM (Fig. 2a). The drug resistance index (RI) is 25.60, calculated by the ratio of IC50 of doxorubicin-resistant MCF-7 cells and IC50 of MCF-7 cells. A chemo-resistant model with RI of 3 or more is considered a successful establishment. Then ABC transporters proteins were detected by western blotting. Results show that P-gp expression of doxorubicin-resistant MCF-7 cells is different from that of MCF-7 cells despite of absence or presence of doxorubicin. However, the protein expression levels of MRP1 and BCRP1 are not apparent both in MCF-7 cell line and doxorubicin-resistant MCF-7 cell line, even in the presence of doxorubicin (Fig. 2b). Furthermore, flow cytometry results confirm that the P-gp expression level of doxorubicin-resistant MCF-7 cells is much higher than that of MCF-7 cells, even in the presence of doxorubicin (Fig. 2c).

### Effects of bioactive constituents of Rhizoma Curcumae on P-gp protein expression in doxorubicin-resistant MCF-7 breast cancer cells

**Fig. 3:**
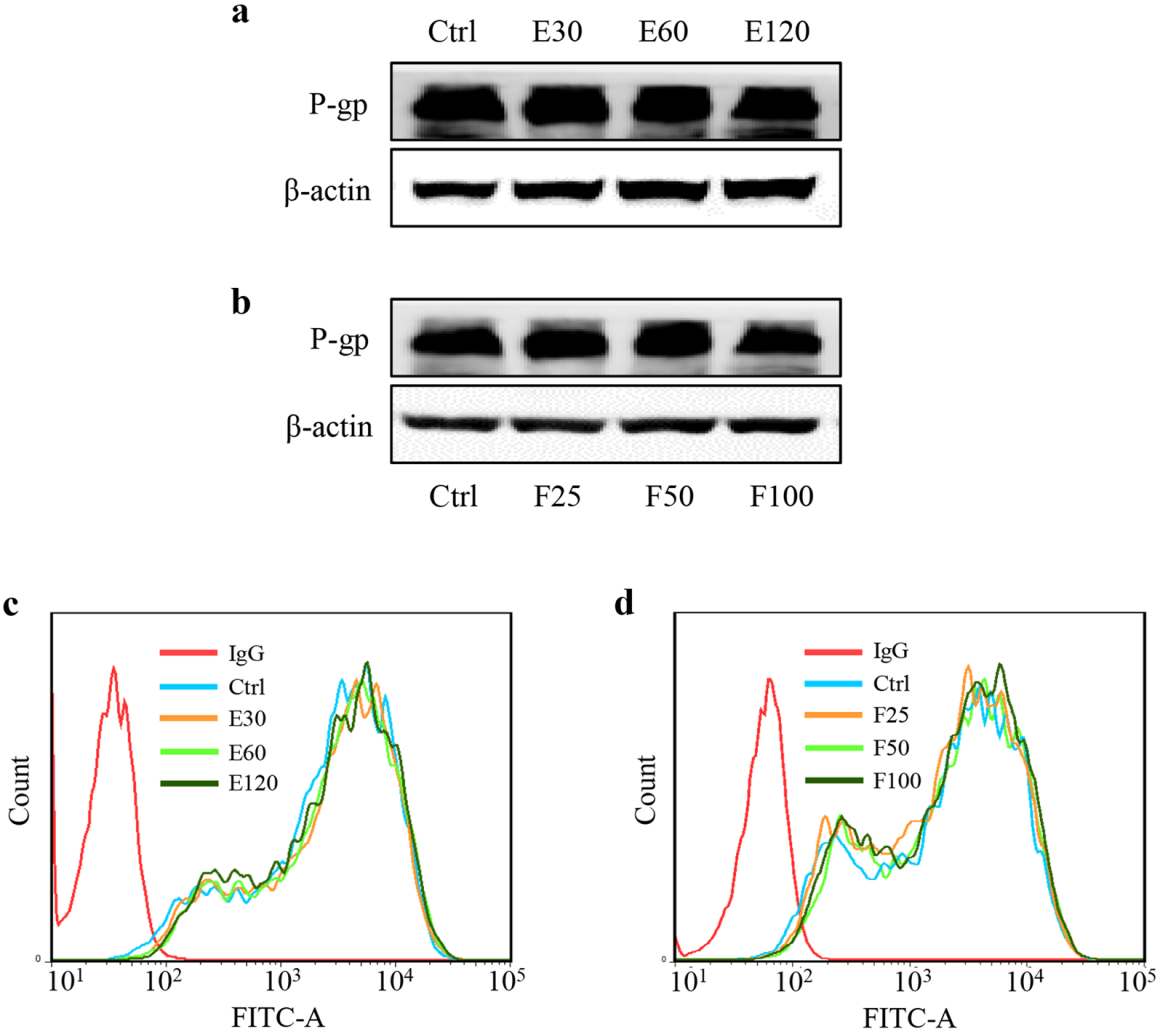
Effects of bioactive constituents of Rhizoma Curcumae on P-gp protein expression in doxorubicin-resistant MCF-7 cells. Cells were treated with different concentrations of essential oil (E; µg/mL) and furanodiene (F; µM) for 24 h, compared with the control (Ctrl). **a**, **b** Protein expression was evaluated using western blotting assay. **c**, **d** Alterations of P-gp expression were confirmed by FITC-P-gp antibody staining using flow cytometry. Data were representative of at least three independent experiments

Protein expression was assessed with western blotting, accompanied by FITC-P-gp staining assays. Different concentrations of essential oil (E30, E60, and E120 are 30, 60, and 120 μg/mL of essential oil, respectively) or furanodiene (F25, F50, and F100 are 25, 50, and 100 μM of furanodiene, respectively) do not show any inhibitory effects on P-gp expression, as shown in Fig. 3a, b. Meanwhile, FITC-P-gp staining assay using flow cytometry show that when compared with the red histogram of isotype control IgG, there are no any significant alterations in P-gp expression after treatment of essential oil or furanodiene at the indicated concentrations. That means P-gp protein expression cannot be affected by essential oil or furanodiene in doxorubicin-resistant MCF-7 cells, as shown in Fig. 3c, d.

### Effects of bioactive constituents of Rhizoma Curcumae on P-gp function in doxorubicin-resistant MCF-7 breast cancer cells

**Fig. 4:**
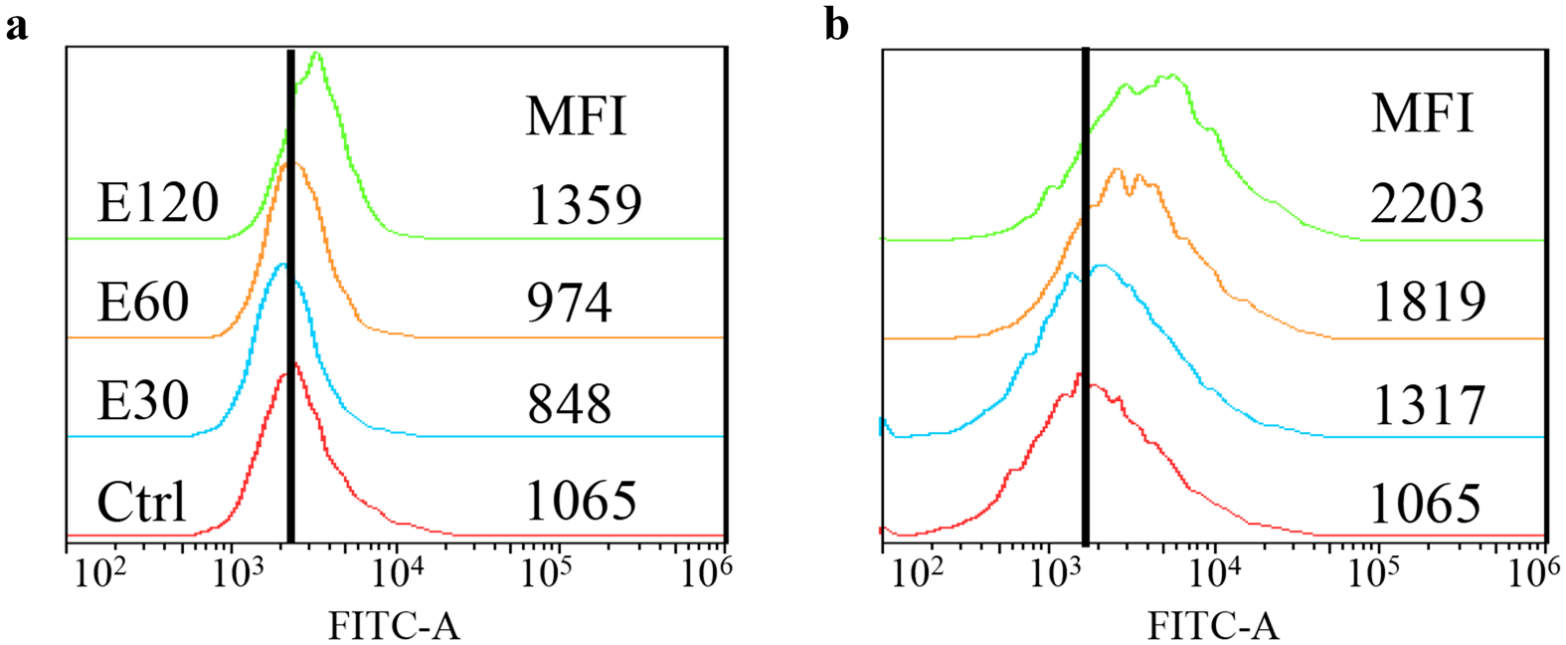
Effect of essential oil on P-gp function in doxorubicin-resistant MCF-7 cells. P-gp function evaluation was performed by a 30-min incubation of **a** Rhodamine 123 and **b** Calcein AM using flow cytometry after 1-h treatment of essential oil (E; µg/mL). Ctrl stands for control. Data were representative mean fluorescence intensity (MFI) of at least three independent experiments

To further investigate influence of bioactive constituents on P-gp function, Rhodamine 123 and Calcein AM uptake assay were employed. After 1-h pre-treatment of essential oil or furanodiene, 30-min incubation with Rhodamine 123 or Calcein AM, fluorescence alterations were determined. Results show that 120 μg/mL of essential oil induces minor enhancements with mean fluorescence intensity (MFI) of 1359 and 2203, represented by P-gp-transported Rhodamine 123 (Fig. 4a) and the intracellular Calcein (Fig. 4b). It indicates that essential oil exerts a slight inhibitory effect on P-gp function in doxorubicin-resistant MCF-7 cells.

**Fig. 5:**
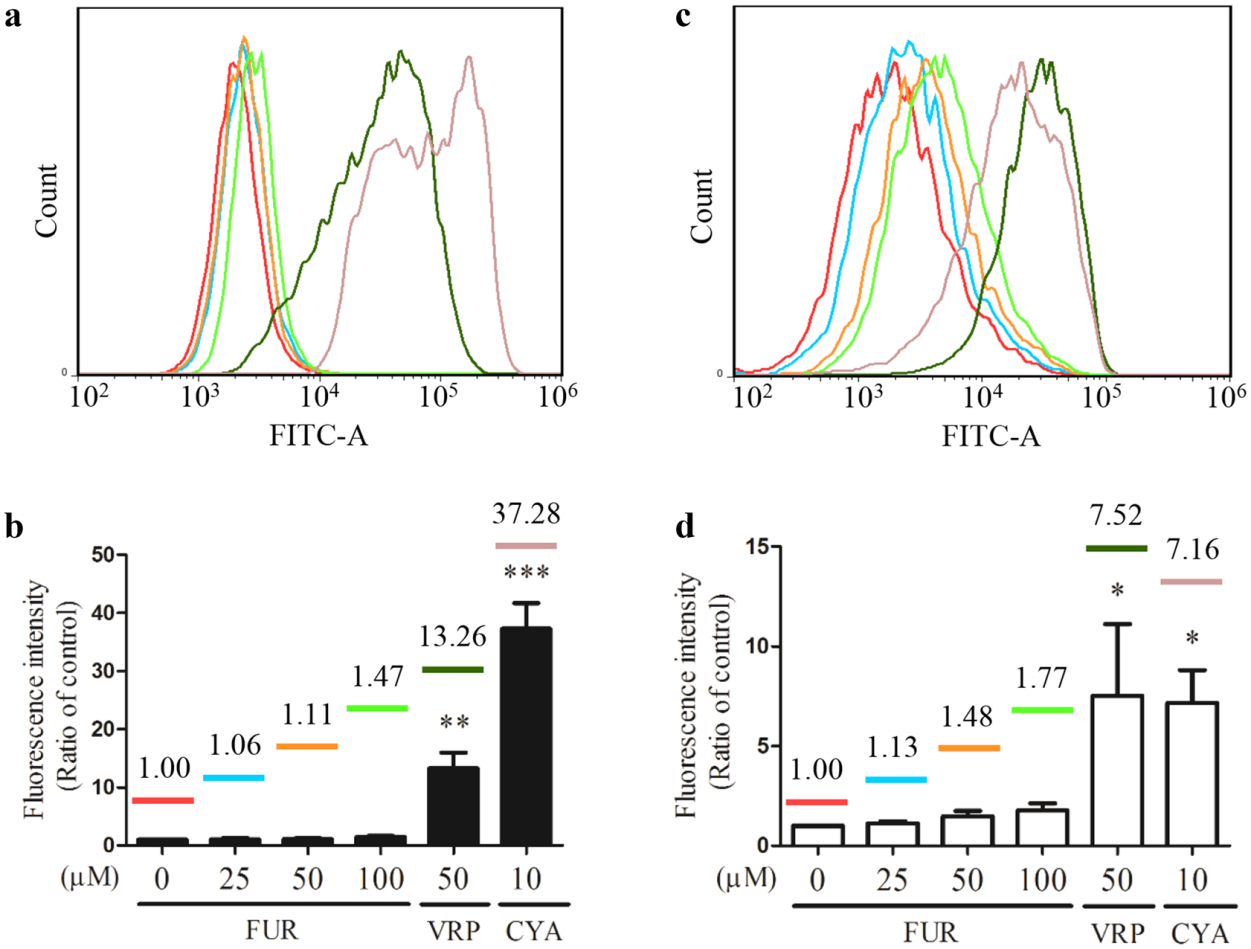
Effect of furanodiene (FUR) on P-gp function in doxorubicin-resistant MCF-7 cells. P-gp function evaluation was performed by a 30-min incubation of Rhodamine 123 (**a**) and Calcein AM (**c**) using flow cytometry after 1-h treatments with furanodiene (FUR), verapamil (VRP), and cyclosporine A (CYA). The corresponding statistical result (**b**, **d**) was shown with VRP and CYA as positive controls. Data were expressed as mean ± S.E.M. *P < 0.05, **P < 0.01, and ***P < 0.001 vs. negative control

Regarding the potential effect of furanodiene on P-gp function, verapamil (VRP) and cyclosporine A (CYA) were used as the positive controls, both in Rhodamine 123 uptake assay and Calcein AM assay. In Rhodamine 123 uptake assay, compared with the remarkable increases (13.26-fold VRP and 37.28-fold CYA) induced by the positive controls, 1.47-fold increasement was observed after 100-μM furanodiene treatment (Fig. 5a, b). Similarly, in Calcein AM assay, compared with the remarkable increases (7.52-fold VRP and 7.16-fold CYA) induced by the positive controls, 1.77-fold increasement was observed after 100-μM furanodiene treatment (Fig. 5c, d).

Collectively, essential oil and furanodiene, both exert a mild inhibitory effect on efflux activity of P-gp in doxorubicin-resistant MCF-7 cells.

### The enhancement of furanodiene on doxorubicin uptake in doxorubicin-resistant MCF-7 breast cancer cells

**Fig. 6:**
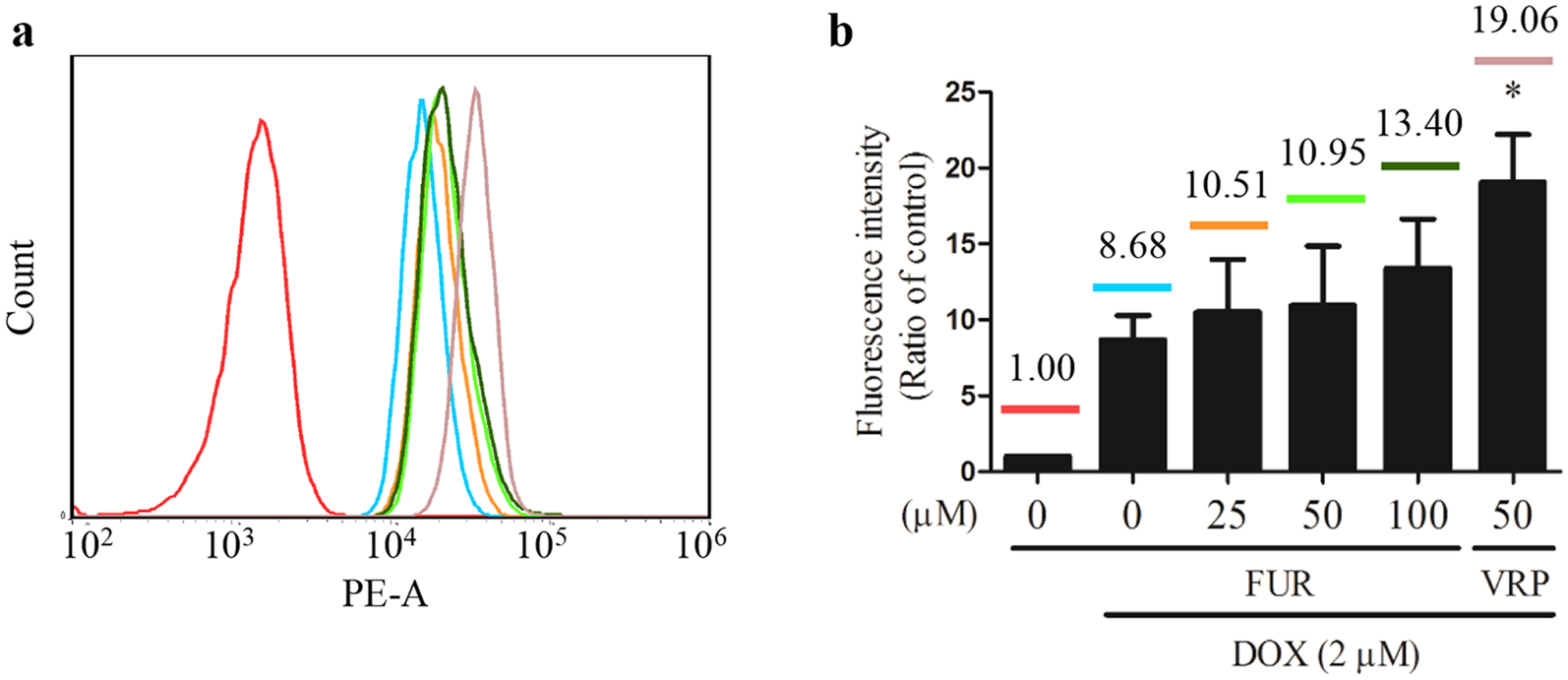
Effect of furanodiene (FUR) on cellular uptake of doxorubicin in doxorubicin (DOX)-resistant MCF-7 cells. **a** After explosion to furanodiene (FUR) or verapamil (VRP) in the presence of doxorubicin (DOX) for 2 h, doxorubicin uptake was analyzed using flow cytometry. **b** Statistical result of **a**. Data shown were expressed as mean ± S.E.M. *P < 0.05 vs. negative control

Doxorubicin uptake assay was performed to further confirm the regulatory effect of furanodiene on P-gp function. Results show that the positive drug verapamil (50 µM) can significantly increase doxorubicin uptake, represented by 19.06-fold fluorescence intensity compared with control. Meanwhile, furanodiene at the indicated concentrations slightly increases doxorubicin uptake without significance (Fig. 6a, b), which indicates that furanodiene may not be a specific inhibitor of ABC transporter protein as verapamil.

### Effects of bioactive constituents of Rhizoma Curcumae on viability of doxorubicin-resistant MCF-7 breast cancer cells

**Fig. 7:**
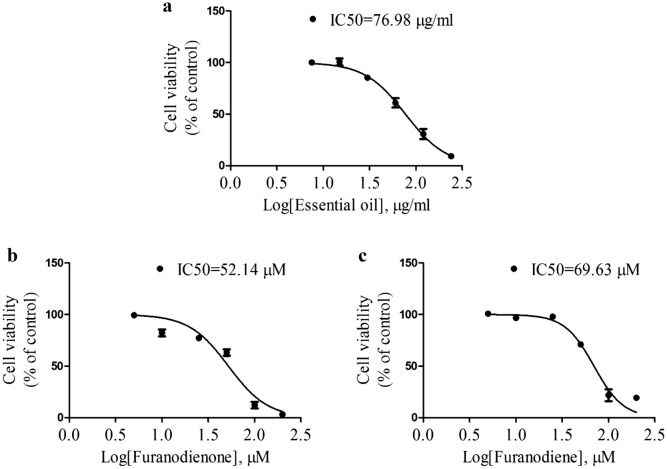
Effects of bioactive constituents of Rhizoma Curcumae on viability of doxorubicin-resistant MCF-7 cells. Cells were treated with different concentrations of **a** essential oil, **b** furanodienone, and **c** furanodiene for 48 h. Cell viability was tested using MTT assay. Data shown were expressed as mean ± S.E.M

To investigate the effect of Rhizoma Curcumae on viability of chemo-resistant cancer cells, doxorubicin-resistant MCF-7 cells were exposed to essential oil, furanodienone, and furanodiene. Cell viability was tested by MTT assay after a 48-h treatment. Results show that essential oil, furanodienone, and furanodiene display powerful inhibitory effects on viability of doxorubicin-resistant MCF-7 cells, with IC50 values of 76.98 µg/mL (Fig. 7a), 52.14 µM (Fig. 7b), and 69.63 µM (Fig. 7c), respectively.

### Combined effects of furanodiene and doxorubicin on viability of doxorubicin-resistant MCF-7 breast cancer cells

**Fig. 8:**
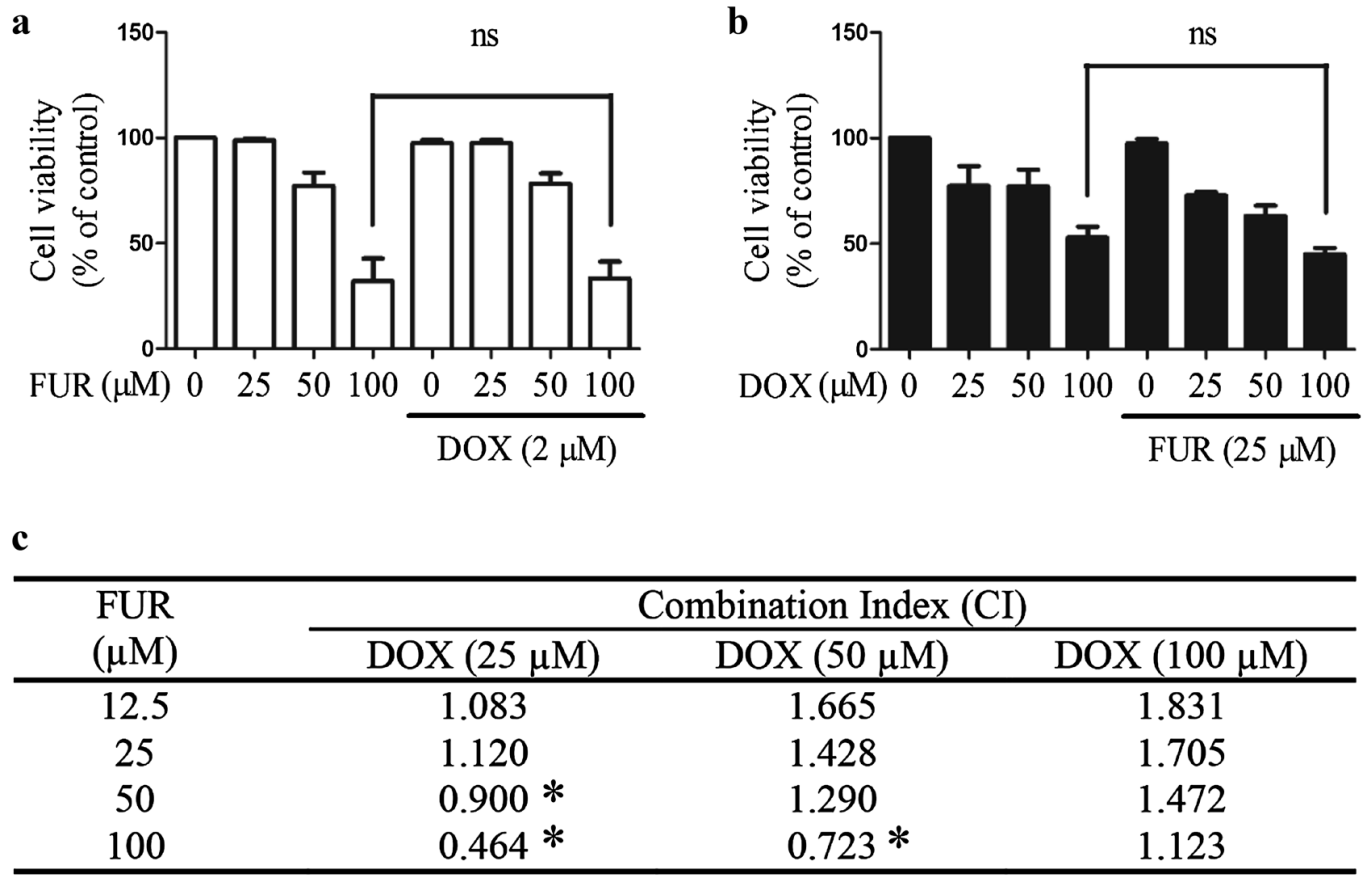
Combined effects of furanodiene (FUR) and doxorubicin (DOX) on viability of DOX-resistant MCF-7 cells. **a** Cells were treated with different concentrations of furanodiene (FUR; 0–100 µM) in the presence or absence of doxorubicin (DOX; 2 µM) for 24 h. **b** Cells were treated with different concentrations of DOX (0–100 µM) in the presence or absence of FUR (25 µM) for 24 h. Cell viability was assessed using MTT assay. **c** The results were analyzed using CalcuSyn program (Biosoft), based on Chou–Talalay method. Data shown were expressed as mean ± S.E.M. ns stands for not significant. *, synergism

Combined effects of furanodiene and doxorubicin on the viability of doxorubicin-resistant MCF-7 cells were determined after 24 h of treatment. Figure 8a, b show that the drug treatment alone (furanodiene or doxorubicin at concentrations of 25, 50, and 100 µM) dose-dependently inhibits the viability of doxorubicin-resistant MCF-7 cells. Doxorubicin (2 µM) and furanodiene (25 µM) are selected in subsequent experiments on account of non-toxic concentrations, as shown in Fig. 8a, b. However, there is no enhancement on sensitivity observed for furanodiene (Fig. 8a) or doxorubicin (Fig. 8b), no significance even at the highest concentration (100 µM of furanodiene or doxorubicin). To further assess the interaction of furanodiene and doxorubicin, cell viability results were analyzed using CalcuSyn program (Biosoft, Ferguson, MO), based on Chou–Talalay method. The combination index (CI) less than one is defined as synergism, while the CI greater than one is defined as antagonism. As shown in Fig. 8c, a synergistic inhibitory effect on the viability of doxorubicin-resistant MCF-7 cells is found when high concentrations of furanodiene combined with low concentrations of doxorubicin; On the contrary, drug antagonism occurs when low concentrations of furanodiene combined with high concentrations of doxorubicin.

## Discussion

Growing evidence claim that Rhizoma Curcumae possesses anti-cancer activity mainly due largely in part to the essential oil and the bioactive components as well, containing curcumin, β-elemene, germacrone, furanodiene, furanodienone, and so on. However, the anti-cancer activities of Rhizoma Curcumae against chemo-resistant cancer cells are not clear yet. Therefore, we reviewed the anti-cancer effects of bioactive constituents of Rhizoma Curcumae against chemo-resistant cancer cells. Collectively, some reports demonstrated that Rhizoma Curcumae extract [19], curcumin [36], β-elemene [37], and germacrone [23] exhibit anti-cancer activities in chemo-resistant cancer cells. However, to the best of our knowledge, there are still research gaps in the exact mechanisms of essential oil, furanodiene, and furanodienone on chemo-resistance. Therefore, our present study was designed to investigate the detailed anti-cancer effects of essential oil, furanodiene, and furanodienone against doxorubicin-resistant MCF-7 breast cancer cells.

Firstly, we identified whether these ingredients are specific inhibitors of ABC transporters or not. Unexpectedly, essential oil and furanodiene cannot affect P-gp expression and slightly inhibit P-gp activity. Accordingly, the underlying mechanisms of Rhizoma Curcumae on chemo-resistance improvement may not be limited to the ABC transporters. Afterwards, our findings show that essential oil, furanodienone, and furanodiene display powerful inhibitory effects on viability of doxorubicin-resistant MCF-7 cells. The results clarified that these ingredients also have anti-cancer activities in chemo-resistant cancer cells. Meanwhile, furanodiene, the potential bioactive compound, was confirmed to induce extrinsic and intrinsic apoptosis through altering mitochondrial function via AMPK-dependent and NF-κB-independent pathways in doxorubicin-resistant MCF-7 cells [32, 38].

According to our previous study, ERα-negative MDA-MB-231 cells are much more sensitive to furanodiene than ERα-positive MCF-7 cells. Therefore, we concluded that furanodiene could significantly increase the efficacy of tamoxifen in ERα-positive breast cancer cells [31]. Therefore, we make an inference from these results that furanodiene may enhance the efficacy of non-steroidal agents in ERα-negative breast cancer cells, instead of in ERα-positive breast cancer cells. Considering these findings mentioned above, we presumed that furanodiene could significantly enhance the efficacy of doxorubicin in ERα-negative and ERα-low expression breast cancer cells [39]. That is why we proposed the subsequent study evaluating the anti-breast cancer activities of furanodiene in combined with doxorubicin on doxorubicin-resistant breast cancer cells. Interestingly, it is actually observed that high concentrations of furanodiene combined low concentrations of doxorubicin exhibit synergistic inhibitory effects on the viability of doxorubicin-resistant MCF-7 cells, and low concentrations of furanodiene combined with high concentrations of doxorubicin exhibit antagonism.

## Conclusions

Overall, even though essential oil and furanodiene are not the specific inhibitors of ABC transporters, these ingredients still display powerful inhibitory effects on viability of doxorubicin-resistant breast cancer cells. The present study not only indicated Rhizoma Curcumae being a promising natural agent for cancer adjuvant therapy in the future, but also filled the gap of the researches on anti-cancer activities and corresponding mechanisms of Rhizoma Curcumae in chemo-resistant cancer cells.

## Additional file

**Additional file 1.** Minimum Standards of Reporting Checklist.

## Abbreviations

DOX: doxorubicin
FUR: furanodiene
VRP: verapamil
CYA: cyclosporine A
MTT: 3-[4, 5-Dimethyl-2-thiazolyl]-2, 5-diphenyltetrazolium bromide
CI: combination index
ABC transporters: ATP-binding cassette transporters
P-gp: P-glycoprotein 1
MRP1: multidrug resistance-associated protein 1
ABCG2: ATP-binding cassette sub-family G member 2
FBS: fetal bovine serum
PBS: phosphate-buffered saline
PS: penicillin–streptomycin
RIPA: radioimmunoprecipitation assay
